# Expression of extraocular *opsin* genes and light-dependent basal activity of blind cavefish

**DOI:** 10.7717/peerj.8148

**Published:** 2019-12-17

**Authors:** Noah Simon, Suguru Fujita, Megan Porter, Masato Yoshizawa

**Affiliations:** 1 Department of Biology, University of Hawai’i at Mānoa, Honolulu, Hawai’i, United States of America; 2 Leonard Davis School of Gerontology, University of Southern California, Los Angeles, CA, United States of America; 3 Department of Biological Sciences, University of Tokyo, Tokyo, Japan

**Keywords:** Hypogean, Swimming burst, Masking, Visual sensation

## Abstract

**Background:**

Animals living in well-lit environments utilize optical stimuli for detecting visual information, regulating the homeostatic pacemaker, and controlling patterns of body pigmentation. In contrast, many subterranean animal species without optical stimuli have evolved regressed binocular eyes and body pigmentation. Interestingly, some fossorial and cave-dwelling animals with regressed eyes still respond to light. These light-dependent responses may be simply evolutionary residuals or they may be adaptive, where negative phototaxis provides avoidance of predator-rich surface environments. However, the relationship between these non-ocular light responses and the underlying light-sensing Opsin proteins has not been fully elucidated.

**Methods:**

To highlight the potential functions of opsins in a blind subterranean animal, we used the Mexican cave tetra to investigate opsin gene expression in the eyes and several brain regions of both surface and cave-dwelling adults. We performed database surveys, expression analyses by quantitative reverse transcription PCR (RT-qPCR), and light-dependent locomotor activity analysis using pinealectomized fish, one of the high-opsin expressing organs of cavefish.

**Results:**

Based on conservative criteria, we identified 33 opsin genes in the cavefish genome. Surveys of available RNAseq data found 26 of these expressed in the surface fish eye as compared to 24 expressed in cavefish extraocular tissues, 20 of which were expressed in the brain. RT-qPCR of 26 opsins in surface and cavefish eye and brain tissues showed the highest opsin-expressing tissue in cavefish was the pineal organ, which expressed exo-rhodopsin at 72.7% of the expression levels in surface fish pineal. However, a pinealectomy resulted in no change to the light-dependent locomotor activity in juvenile cavefish and surface fish. Therefore, we conclude that, after 20,000 or more years of evolution in darkness, cavefish light-dependent basal activity is regulated by a non-pineal extraocular organ.

## Introduction

Opsins are a family of seven-transmembrane receptor proteins that are able to form a photosensitive complex by binding a molecule of the chromophore retinal (Findlay & Pappin, 1986; Porter et al., 2012). The Opsin family is composed of visual Opsins, expressed in retinular photoreceptors of the eye, and non-visual Opsins expressed in both ocular and extraocular tissues. For example, a study of zebrafish found 42 *opsins* in the genome, with expression in the brain, eye, gut, heart, liver, muscle, pineal, skin and testis (Davies et al., 2015). While functional roles of visual Opsins have been well characterized, functional roles for non-visual Opsins remain largely unknown, with some exceptions. One example is *exo-rhodopsin (exo-rod),* which is expressed in the pineal and is involved in the regulation of circadian rhythms (Pierce et al., 2008). Others are cone opsins expressed in the skin of tilapia that regulate the aggregation and dispersion of pigment granules in melanophores and erythrophores (Chen, Robertson & Hawryshyn, 2013), and brain opsins (possibly Melanopsin—Opn4a) regulating behavioral phototaxis/-kinesis in zebrafish larvae (Fernandes et al., 2012).

The research by Fernandes et al. (2012) on Melanopsin expressed in zebrafish brain tissue highlighted that extraocular opsins have the potential to regulate non-circadian animal behaviors such as light-seeking behavior. The links between extraocular photoreceptors and non-circadian behavior have also been suggested from studies of fossorial and cave-dwelling animals. In blind cave salamanders, negative phototaxis is possibly mediated through degenerated eyes and/or the pineal (Kos et al., 2001; Schlegel, Steinfartz & Bulog, 2009). In fish, negative phototaxis in blind Somalian cavefish has been suggested to be linked to the expression of opsin proteins in the brain, possibly by tmt-opsin, melanopsin, rhodopsin or exo-rhodopsin (Berti & Ercolini, 1975; Cavallari et al., 2011; Tarttelin et al., 2012; Calderoni et al., 2016). However, none of these studies have shown which type of opsin genes are still expressed in blind cave-adapted morphs in regressed eyes and the brain, and which non-visual Opsins potentially keep their functions. Here we used the Mexican blind cavefish, *Astyanax mexicanus*, to perform an initial investigation for these questions.

*A. mexicanus* is a well-studied evolutionary model for cave adaptation. This species includes 30 cave-dwelling populations (i.e., cavefish), which have evolved a number of morphological and physiological traits associated with dark environments such as regressed eyes and body pigment (Mitchell, Russell & Elliott, 1977; Wilkens, 1988; Espinasa et al., 2018), and also includes surface-dwelling populations which have functional eyes and body pigments (i.e., surface fish). The severe regression of the Mexican cavefish binocular eyes has been well characterized, including loss of the rhodopsin-expressing retinal cells (Yamamoto & Jeffery, 2000). Despite regressed eyes, juvenile and adult cavefish still display light-dependent basal locomotor activity, i.e., increased activity in well-lit environments relative to dark (Duboué, Keene & Borowsky, 2011; Beale et al., 2013; Moran, Softley & Warrant, 2014; Yoshizawa et al., 2015). Note, this basal locomotor activity is not directional but depends on ambient intensity of light, i.e., the adult cavefish do not show either positive or negative phototaxis but their average swimming speed increases (Kowalko et al., 2013). In cavefish, two strands of evidence suggest that the gene-based circadian clock does not seem to regulate this light-dependent basal activity. First, the circadian clock in Mexican cavefish is attenuated; the light-responding *per2* gene is constitutively expressed as if cavefish are experiencing constant light, irrespective of the light environment (Beale et al., 2013). Second, in adult Mexican cavefish (e.g., Pachón cavefish) there is no circadian locomotor activity during 24 h dark conditions following a 12:12 h light-dark training period (Beale et al., 2013; Moran, Softley & Warrant, 2014). In other independently arisen cave populations (e.g., Tinaja cave population), a weakly trainable circadian clock appears to have been retained, i.e., light-dark cycles make expression levels of circadian clock genes oscillate and these oscillations continue briefly after the removal of the light-dark cycle (∼1 day) (Caballero-Hernández et al., 2015; Carlson & Gross, 2018; Carlson et al., 2018). However, the majority of light-dependent basal locomotor activity seems to be regulated by “masking”, where the ambient light overwrites 24 h-rhythmic circadian behaviors (Rietveld, Minors & Waterhouse, 1993; Caballero-Hernández et al., 2015). The molecular mechanism for masking in fish is largely unknown (but see mouse studies in Panda et al., 2003; Van Gelder, 2003). Also, the opsins and light detecting tissues regulating this light-dependent basal locomotor activity have not been well studied.

To resolve the relationship between Opsins and cavefish light-dependent basal activity, we first surveyed the *A. mexicanus* genome for *opsin* family genes to characterize the full set of possible extraocular photopigments. There is no comprehensive study for how many opsin family genes are coded in the *A. mexicanus* genome, and whether they regulate the light-dependent locomotion in cavefish (but see the genomic study by McGaugh et al., 2014). Classic studies provided a strong base for this study: Yokoyama & Blow (2001), Yokoyama & Yokoyama (1993) and Yokoyama et al. (1995) reported that *A. mexicanus* cavefish have accumulated null mutations in some of the visual opsins including rhodopsin, which forms the visual pigment used for dim-light vision in vertebrates. Once identified, we investigated the extraocular roles of *opsin* genes by surveying expression levels in adult fish tissues. Such Opsins regulate circadian rhythm (exo-rhodopsin), and/or act as photoisomerases to recover 11-cis retinal from the 11-trans form. We were particularly interested in extra-ocular opsin expression in the post-larval (i.e., juvenile) stage because, at this point of development, fish are still small in size to be handled (1.0–1.5 cm in the standard length) but already show adult-type phenotypes, i.e., the cavefish juvenile eyes have mostly degenerated and fish exhibit increased basal activity in response to light. Expression levels for a representative set of the *opsin* genes in the genome were quantified by using quantitative reverse transcription polymerase chain reaction (RT-qPCR) for the eyes and four brain regions of surface and cavefish. Based on these studies, where pineal showed the highest *opsin* expression in cavefish, we then tested the role of pineal photoreceptors in the light-dependent activity shift by a pinealectomy experiment. A prior study in *A. mexicanus* demonstrated that the light-dependent activity shift emerges as early as 21 days post fertilization (juvenile stage: see ‘Materials and Methods’) (Duboué, Keene & Borowsky, 2011). During this juvenile stage, interestingly, both pinealectomized surface fish and cavefish retained the light-dependent activity shift. From our study, we concluded that cavefish retain the expression of many non-visual opsins and that the light-dependent activity shift is regulated by a non-visual, non-pineal, extraocular light sensing tissue.

## Materials & Methods

### Fish maintenance and rearing

*Astyanax mexicanus* surface fish used in this study were laboratory-raised descendants of original collections made in Balmorhea Springs State Park, Texas. Cavefish were laboratory-raised descendants of fish collected from Cueva de El Pachón (Pachón cavefish) in Tamaulipas, Mexico (Mitchell, Russell & Elliott, 1977). They were originally collected in the field between 2000 and 2001 by Dr. William R. Jeffery’s research group, and both morphs have passed approximately 10–15 generations in the laboratory. Fish (surface fish and Pachón cave populations) were housed in the University of Hawai’i at Mānoa *A. mexicanus* facility with temperatures set at 21 °C  ± 0.5 °C for rearing, 27 °C  ± 1 °C for behavior experiments and 25 °C  ± 0.5 °C for breeding (Borowsky, 2008; Yoshizawa, Ashida & Jeffery, 2012; Elipot et al., 2014; Yoshizawa et al., 2015). Water used to house the fish was conditioned to a pH of 6.8–7.0 and a conductivity of 600–800 µS. Lights were maintained on a 13:11 light/dark cycle (Yamamoto et al., 2003; Borowsky, 2008; Elipot et al., 2014). For rearing and behavior experiments, light intensity was maintained between 30 and 100 Lux. Fish husbandry was performed as previously described (Yamamoto et al., 2003; Borowsky, 2008; Yoshizawa, Ashida & Jeffery, 2012). Adult fish were fed a mixed diet to satiation two times daily starting 3 h after the lights came on (Zeitgeber time 3 or ZT3) and ZT8 (TetraColor Tropical Fish Food Granules and TetraMin Tropical Fish Food Crisps, Tetra, Blacksburg, VA; Jumbo Mysis Shrimp, Hikari Sales USA, Inc., Hayward, CA). All fish in the behavioral experiments were between 1.2 and 1.5 cm in standard length and between 6 and 16 weeks old. We use the stage terms as follows: the embryonic stage (before hatching): 0–24 h post fertilization (hpf); the larval stage without obvious reproductive organs: 24 hpf–1 month post fertilization (mpf); the juvenile stage with developing reproductive organs (1 mpf–4 mpf); and young adult stage (4 mpf–12 mpf). All fish care and experimental protocols are approved under the Animal Care & Use Committee at University of Hawai’i (IACUC 17-2560).

### Genomic and transcriptomic survey of *opsin* family genes

Consensus sequences were generated from zebrafish, tilapia, and medaka for known opsin genes (zebrafish opsin accession numbers shown in Table S1) using Clustal Omega (Sievers et al., 2011) and Geneious software version 9.2 (https://www.geneious.com; Biomatters limited, Auckland, New Zealand) (Kearse et al., 2012). Consensus sequences were queried against the *A. mexicanus* genome and cDNA database (AstMex 1.0.2, genebuild 2016 spring) (Yates et al., 2016) with the local BLAST server under the environment of SequenceServer 1.0.6 (http://www.sequenceserver.com/). In the set of genes identified, there were many genes that included sequences from different sources that varied by indels or single base pair identity (see Table S1). For example, for the *rhod* gene our methods identified three sequences: one from the NCBI GenBank cDNA repository (U12328.1), one from the Ensembl prediction (ENSAMXG00000026346, based on 100 bp short-read sequences of whole-genome and transcriptome data), and a predicted sequence from the result of BLAST searches in *A. mexicanus* Sequence Read Archive (SRA) repositories at NCBI (Transcriptome); these sequences vary by 61–100% identity, mainly due to indels, and potentially indicate methodologically-introduced sequence errors (Table S1). In this case, the NCBI cDNA repository has the most evidence from wet-experiments, followed by the NCBI and Ensembl annotations based on either transcriptomic or genomic short-read sequences (AstMex 1.0.2, genebuild at July 2016), and finally the predicted sequence based on SRA BLAST alignment. Similar to this example, for *opsin* genes where sequences from different sources contain indel or base pair variations, we chose a conservative approach by choosing to use the single sequence with the most support from experimental evidence, using the following criteria in ranked order of preference: (1) cDNA repository sequence, (2) the latest predicted sequence in either NCBI or Ensembl, (3) the homemade-predicted sequence based on SRA BLAST alignment. Indels were dropped from further analysis because of frequent inconsistencies between databases.

We then surveyed *opsin* expression by querying predicted cDNA sequences for each *opsin* against available NCBI Sequence Read Archive (SRA) databases using NCBI BLASTn and MegaBLASTn for the following published samples: (1) embryonic to larval stage tissues (from 10 hours-post-fertilization (hpf) to 36 hpf between surface and cavefish; BioProject ID: PRJNA258661) (Stahl & Gross, 2015; Stahl & Gross, 2017; Yoshizawa et al., 2018); (2) adult surface and cavefish whole body databases (BioProject ID: PRJNA183542) (Gross et al., 2013); and (3) individual *A. mexicanus* tissues (Table 1) (BioProject ID: PRJNA177689) (McGaugh et al., 2014). For individual tissue transcriptomes (PRJNA177689), since these were raw data consisting of 100 bp fragments, the MegaBLAST results were analyzed visually to assess whether the aligned 100 bp fragments covered the entire queried cDNA (993–5,052 bp in length depending on *opsin* genes). More than two thirds coverage of queried cDNA by 100 bp SRA sequences (each 100 bp sequence showed 100% identity in alignments) was considered a positive hit. Tissue transcriptome data was available for surface fish eye and Pachón cavefish brain, skin, nasal, kidney, liver, heart, and muscle tissues. MegaBLAST results of the tissue transcriptomes (Table 1) indicated that 26 opsins were expressed in the *A. mexicanus* surface fish eye (note: no transcriptome for cavefish eye in PRJNA177689), with a subset of 20 of these expressed in the cavefish brain. We thus used these representative 26 opsins to perform real-time quantitative reverse transcription PCR (RT-qPCR) assays and analyzed their expression levels in eyes and brain regions.

**Table 1:** Opsin expression in *A. mexicanus* surface fish eye and 7 Pachón cavefish tissues based on BLAST results against published transcriptome data.

	Eye (surface)	Brain (cave)	Skin (cave)	Nasal (cave)	Kidney (cave)	Liver (cave)	Heart (cave)	Muscle (cave)
*rhod*	+	+	+	+	+	+	+	+
*lws*	+	+	–	–	+	+	–	–
*mws*	+	–	–	–	–	–	–	–
*g101*	+	–	–	–	–	–	–	–
*g103*	+	–	+	+	+	–	–	–
*sws2*	+	–	–	–	–	–	–	–
*rhol*	+	–	–	–	–	–	–	–
*exo-rod*	+	+	–	+	–	–	–	–
*vab*	+	+	–	–	+	–	–	–
*vaa*	+	+	–	–	–	–	–	–
*parapina*	–	–	–	+	–	–	–	–
*parapinb*	+	–	–	–	–	–	–	–
*parietopsin*	–	–	–	–	–	–	–	–
*tmt1a*	–	+	+	–	–	–	–	–
*tmt1b*	+	+	+	+	+	–	–	–
*tmt2a*	+	+	+	+	–	–	–	–
*tmt2b*	+	+	–	–	–	–	–	–
*tmt3a*	–	+	–	–	–	+	–	–
*opn3*	+	+	+	+	+	–	–	–
*opn6a*	+	+	–	–	–	–	–	–
*opn6b*	+	–	–	–	–	+	–	–
*opn7a*	–	+	–	–	+	–	–	–
*opn7d*	+	+	–	–	–	–	–	–
*opn9*	+	+	+	–	–	+	–	–
*rgra*	+	+	+	+	+	+	+	–
*rgrb*	+	–	–	–	–	+	–	–
*rrh*	+	+	+	+	+	–	–	–
*opn5*	+	+	+	–	+	–	–	–
*opn4m1*	–	–	–	–	–	–	–	–
*opn4m2*	+	+	–	–	–	–	–	–
*opn4m3*	+	+	–	–	–	–	+	–
*opn4x1*	+	–	–	–	–	–	–	–

**Notes.**

‘ +’ and ‘–’ are based off the MegaBLAST search hit on NCBI SRA transcriptomic data (BioProject: PRJNA177689). Note that the NIH SRA database (PRJNA177689) only contains surface fish eyes but not cavefish eyes, and cavefish extraocular tissues but not surface fish extraocular tissues.

### Phylogenetic analysis

Amino acid sequences (Table S2) of zebrafish and cavefish opsins were analyzed using Geneious software. GPCR sequences closely related to opsins from humans (Fredriksson, 2003) and *Trichoplax adhaerens* (Srivastava et al., 2008) were used as outgroups (Table S2). Bovine rhodopsin was also included to confirm the alignment of the transmembrane regions. The Transmembrane Prediction Tool plugin (https://www.geneious.com/plugins/transmembrane-prediction-plugin/) was used to annotate the transmembrane regions of each protein. The predicted extracellular regions at the N- and C- termini were trimmed from each sequence in order to include only the more conserved transmembrane regions. Sequences from *A. mexicanus rgrb* and *opn6b* (Ensembl) had only 3 transmembrane regions each and were shorter than the other opsins, suggesting incomplete sequences. Because of this, untrimmed sequences were used for these opsins in the analysis. An alignment was generated using the MUSCLE alignment algorithm. A maximum likelihood tree was generated using the ‘RAxML-HPC2 on XSEDE’ Tool (Stamatakis, 2014) on the CIPRES Science Gateway (Miller, Pfeiffer & Schwartz, 2010). Tree graphics were generated using FigTree software version 1.43 (http://tree.bio.ed.ac.uk/software/figtree/) and edited using Abode Photoshop CC 2018.

### Real time quantitative reverse transcription polymerase chain reaction (RT-qPCR)

**Figure 1:**
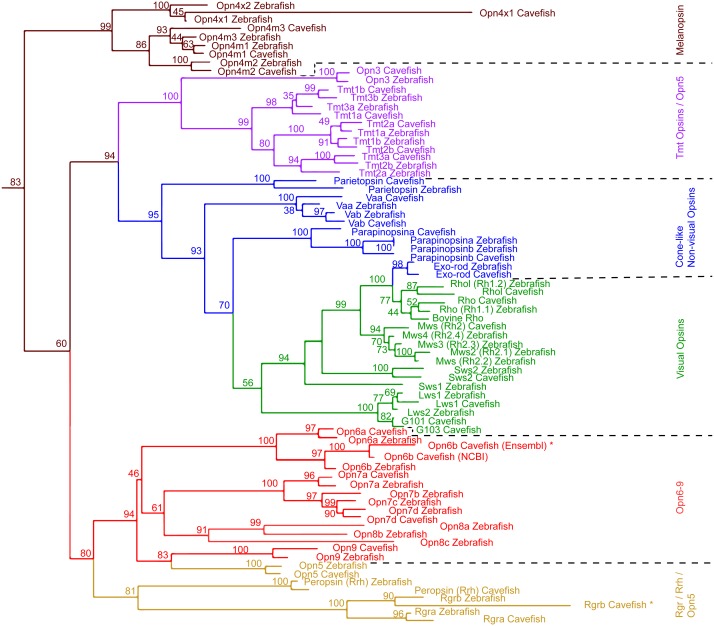
Maximum Likelihood tree of *Astyanax mexicanus* and *Danio rerio* opsins. Maximum likelihood analysis based on MUSCLE alignment of opsin amino acid sequences. The non-transmembrane regions at the N- and C- termini were removed; outgroups not shown. Transcripts that were not full length are indicated by ‘*’.

Two adult surface fish and two adult Pachón cavefish (5 years old, randomly chosen) were euthanized in buffered ice water with 0.5 mg/ml of MS-222 (pH at 7.0; Millipore-Sigma, Burlington, MA) and decapitated in ice-cold PBS. The dissection was performed between 9 am and 11 am (Zeitgeber time 2 and 4) to standardize the potential circadian regulation of opsin expression. Eyes from the two surface fish and degenerated eye cysts from two cavefish were dissected, pooled into 1.5 mL tubes by each morph (two tubes: one for surface fish and another for cavefish), and immediately frozen on dry ice. Brains were removed from the skull and pineal, telencephalon, tectum and cerebellum regions were dissected from each, and each brain region was pooled by morph (Fig. 1). The remaining basal brains were used as ‘deep brain’ samples. The separate brain samples were also immediately frozen on dry ice. Total RNA was extracted from each sample using QIAGEN RNeasyPlus Universal Mini Kit (QIAGEN). To avoid enzymatic inhibition by retinal pigment, the extracted total RNA of surface fish eye was additionally purified through OneStep PCR Inhibitor Removal Kit (Zymo Research, Irvine, CA). To synthesize complementary DNA (cDNA), iScript gDNA Clear cDNA Synthesis Kit (Bio-Rad Laboratories, Hercules, CA, USA) was used with 1 µg of total RNA from each sample in 10 µL reactions following manufacturer’s instructions. RNA and cDNA concentrations were quantified using Qubit 3.0 Fluorometer assay kits (Qubit RNA BR Assay Kit and Qubit ssDNA Assay Kit for total RNA extraction and cDNA synthesis, respectively; ThermoFisher Scientific, Waltham, MA). RNA was then stored at −80 °C; cDNA was stored at −20 °C until used.

Primers for 26 *opsins* and 3 reference housekeeping genes (*b2m, eef2a.1* and *rps18*) were successfully designed using PerlPrimer software (Marshall, 2004). Two primer sets were designed for each gene, each amplifying different regions of the gene, and the better of the two was used for the expression assays (i.e., amplification efficiency between 90–110%, and melt-curve profile showing a single-sharp peak instead of a broad peak or two-peaks; Taylor et al., 2010). The reference genes were selected based on a study in zebrafish showing stable expression across different tissue types (McCurley & Callard, 2008). Primers were designed between 18 and 22 bases long with a melting temperature (Tm) between 58 and 62 °C. Each primer pair was designed to have a Tm difference of less than 1 °C. The PCR-target intervals were designed between 70 to 150 bp, and also, at least one of the primers is selected to overlap an intron splice site by 7 bases (except for single exon genes). Quality checks for the amplification specificities and efficiencies of RT-qPCR primers were performed using a serial dilution of a positive control cDNA library constituted of a mix of equal amounts of cDNA from surface fish eyes and skin tissues, within which most identified opsins are expressed (Table 1). SsoAdvanced Universal SYBR Green Supermix was used for RT-qPCR amplification (Bio-Rad Laboratories). Assays were run on a Bio-Rad CFX96 Touch Real-Time PCR Detection System. Bio-Rad CFX Manager Software version 3.1 was used to analyze RT-qPCR results. To check the primer specificity, we checked melt curve shapes to evaluate if non-specific amplification occurred (see below).

For efficiency checks, we first used the 90–110% efficiency range (Taylor et al., 2010) (Table S3). The following primer sets were outside of this range: *mws, rhol, exo-rod, vab, parapina, tmt1a, tmt1b, tmt2a, tmt2b, tmt3a, opn3, opn7d, opn9, opn5, rrh, opn4m3, opn4x1* (Table S3). The primers for *opn4m2, opn6b,* and *rhod* were also slightly outside of the recommended 90–110% efficiency range (generated by Bio-Rad CFX Manager) (Table S3). These poor scores are likely due to low expression levels of these genes. We carefully evaluated these primers’ amplification capacities by checking (1) whether the primer sets can amplify its product at two or more concentration points of cDNA serial dilution; (2) whether amplification amounts meet approximately “2^*N*^” under *N*-times of PCR cycle; and (3) whether melt curve indicates specific amplification (no non-specific amplification). Only PCR primers passing these criteria were used in this study.

**Figure 2:**
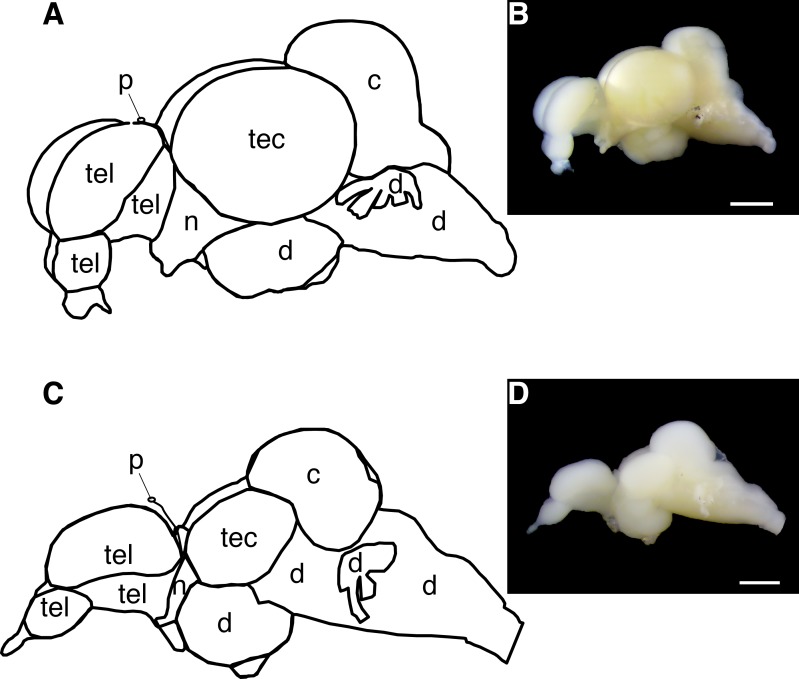
Diagram of the *Astyanax mexicanus* brain showing gross brain regions that were dissected for quantitative reverse transcriptase PCR. (A, B) Surface fish brain. (C, D) Cavefish brain. Abbreviations: tel, telencephalon; tec, tectum; c, cerebellum; d, thalamus; hypothalamus, midbrain and hindbrain; n, optic nerve; p, pineal. Note, opsin expression in cerebellum was not investigated in this study due to the limit of assay capacity (see ‘Materials and Methods’). Bars in pictures: 1.0 mm.

RT-qPCR expression assays were performed separately for: eyes, pineal, tectum, telencephalon, deep brain and skin of surface fish and Pachón cavefish (e.g., Fig. 2). Each reaction was performed with a total volume of 10 µL, using 1 ng of cDNA and a final primer concentration of 500 nM; cycle temperatures and times are shown in Table S4. Each experimental condition was experimentally duplicated (*N* = 2). Expression values were calculated using the 2^−ΔΔ*Cq*^ method (Livak & Schmittgen, 2001) and normalized to a value of 1 for the median data point across all genes and tissues.

All run-to-run differences between RT-qPCR assays were accounted for using an inter-run calibrator sample tissue—a cDNA cocktail of eye and skin—to ensure that the expression differences between opsin genes in this study were a comparable proxy of the genuine differences of the expression levels across genes.

### Pinealectomy

1.5–2.5 days post-fertilization (dpf), both surface fish and cavefish larvae were embedded in 1% agar in conditioned water (approx. 700 µS, pH at 7.0) at 40 °C. Removal of the pineal gland was performed as previously described (Yoshizawa & Jeffery, 2008). Briefly, ∼50 healthy larvae of each group were cleaned by removing debris from the water, and were transferred to a 3.5 cm Petri dish. After removing excess of the fish conditioned water by pipette, larvae were transferred to 5 ml of a 40 °C pre-warmed 1% agar solution and quickly poured into a 40 °C pre-warmed 6 cm Petri dish. To position the larvae on their side, excess agar solution was removed during solidification. The grape cluster-like pineal eye, which sits between the telencephalon and tectum, was removed by an electrically sharpened tungsten needle (wire with a 0.1 mm diameter) after making an opening in the skin. Fish in the control groups were embedded in agar, the skin was removed and a nearby brain region—tectum or telencephalon—was speared, and then freed. Fish were raised as described above and video assayed at 6–16 weeks postfertilization.

### Activity assay in light/dark

Activity of individual fish was recorded inside a custom-built black box chamber (305 × 349 × 1,524 mm) made of a PVC pipe frame covered by a blackout curtain. Each fish was housed in an individual well of a transparent 6-well plate filled with the conditioned water (total 6 individuals in a 6-well plate. see Pinealectomy section). The 6-well plate was placed on top of a custom light box made from 3.2 mm thick white acrylic boards (Min Plastics, Honolulu, HI, USA). The light box was illuminated from below with IR LED lights (Infrared 850 nm 5050 LED Strip Light, Environmental Lights, San Diego, CA, USA). White LED strips were placed in the recording chamber above the fish and maintained on a 13:11 h light:dark timer. Fish were acclimated in the chamber overnight and recorded for 24 h starting approximately 2 h after lights on (ZT-2). Videos were recorded using a USB webcam (LifeCam Studio 1080p HD Webcam, Microsoft, Redmond, WA, USA) fitted with a zoom lens (Zoom 7000, Navitar, Rochester, NY, USA). An IR high-pass filter (Optical cast plastic IR long-pass filter, Edmund Optics Worldwide, Barrington, NJ, USA) was placed between the camera and the lens to block visible light. Video was captured at 15 frames s^−1^ using VirtualDub software (version 1.10.4, http://www.virtualdub.org/) with a vfw codec (https://sourceforge.net/projects/x264vfw/). Fish movements were tracked using SwisTrack 4 software. Data was extracted using custom Perl scripts (v5.10.0) and Excel macros (Microsoft). Fish activity was measures in 10 min bins of total swimming distance. For the day and night basal-activity analysis, we chose the middle 4 h of the day and night (ZT6-10 and ZT19-23, respectively) to exclude the effect of swimming bursts (see ‘Results’) (Burgess & Granato, 2007b).

### Statistics

For statistical comparisons, we performed parametric tests including student’s *t*-tests to compare between surface and cavefish. One-way analysis of variance (ANOVA) was used for analysis of opsin expression levels, post-hoc Tukey-b tests were performed separately for each tissue (eye, pineal, telencephalon, tectum, and deep brain) to identify homogeneous subsets by setting the cutoff alpha as 0.05. Cerebellum tissue was excluded for the expression analysis due to the limit of space in the 96-well PCR plates. Three-way ANOVA was used for factorial analysis of morphs (surface and cavefish), experimental sets (control and pinealectomized), and the lighting condition (light or dark). We then applied a post-hoc test based on Bonferroni adjusted student *t*-test (i.e., *α*_altered_ = 0.05∕(number of tests)) on the comparison between the 4 h light and 4 h dark periods to avoid the period of the swimming burst induced by turning on or off the light (see ‘Result’ section). These calculations were conducted using IBM SPSS 24.0.0.1 software (IBM, Somers, NY, USA) and all statistical scores are available in figure legends and body text.

### Data availability

All analyzed qPCR data are available in the Supplemental Files. Original video data were deposited at Zenodo (http://doi.org/10.5281/zenodo.3533322).

## Results

### *A. mexicanus opsin* repertoire

We surveyed the *Astyanax* Ensembl gene annotation (Ensembl.org; AstMex 1.0.2, genebuild at July 2016) (McGaugh et al., 2014; Yates et al., 2016), and the NCBI genbank repository (gene repository) for opsin genes/transcripts, resulting in 50 partial sequences of *A. mexicanus* opsin genes. In this partial sequence set were many sequences for a single opsin gene from different sources that varied by short indels or single base pair identity (Table S1). For the following studies, we chose the 33 most reliable sequences among these 50 available sequences based on previously published experimental evidence (Table 2 and Table S5) (reliability check, also see the section of ‘Genomic and Transcriptomic Survey of opsin Family Genes’ in the ‘Materials & Methods’). This set of 33 sequences included 8 visual and 6 cone-like non-visual Opsins, 4 Melanopsin and 19 new non-visual Opsins. We first investigated their phylogenetic structure by converting them into amino acid sequences for phylogenetic comparison with 42 zebrafish Opsin sequences (Fig. 1).

**Table 2:** Homologs of Zebrafish *opsins* (Davies et al., 2015) in *A. mexicanus* Databases and Expressions in surface and cavefish.

	Gene name	Zebrafish Gene ID	*A. mexicanus* Gene ID	Surface fish	Cavefish
Visual *opsins*	*rhod*	ENSDARG00000002193	U12328.1 (NCBI)	–	–
	*rhol*	KT008393 (NCBI)	ENSAMXG00000024894	–	–
	*mws1*	KT008394 (NCBI)	ENSAMXG00000001266^*^	–	–
	*mws2*	KT008395 (NCBI)			
	*mws3*	KT008396 (NCBI)			
	*mws4*	KT008397 (NCBI)			
	*sws1*	KT008398 (NCBI)	N/A*	N/A	N/A
	*sws2*	ENSDARG00000017274	AH007939.1 (NCBI)	+	+
	*lws1*	ENSDARG00000044862	XM_007237519.2 (NCBI)	+	+
	*lws2*	ENSDARG00000044861	ENSAMXG00000006368	+	–
	*g101*	N/A	U12024.1 (NCBI)	+	+
	*g103*	N/A	U12025.1 (NCBI)	+	+
Cone-like non-visual *opsins*	*exo-rod*	ENSDARG00000103574	ENSAMXG00000017182	–	+
	*vaa*	KT008402 (NCBI)	ENSAMXG00000009826	–	–
	*vab*	ENSDARG00000054181	XM_007259147.2 (NCBI)	–	–
	*parapinopsina*	KT008404 (NCBI)	XM_007237073.2 (NCBI)	–	–
	*parapinopsinb*	ENSDARG00000044672	ENSAMXG00000007169	–	–
	*parietopsin*	KT008406	ENSAMXG00000010213	–	–
*tmt / opn3*	*tmtops1a*	ENSDARG00000103674	ENSAMXG00000016913	–	–
	*tmtops1b*	ENSDARG00000032246	ENSAMXG00000008135	+	+
	*tmtops2a*	KT008407 (NCBI)	KF737856.1 (NCBI)	–	–
	*tmtops2b*	ENSDARG00000027822	ENSAMXG00000003866	–	+
	*tmtops3a*	ENSDARG00000036460	ENSAMXG00000019922	+	+
	*opn3*	ENSDARG00000052775	XM_007258701.2 (NCBI)	+	+
*opn6-9*	*opn6a*	ENSDARG00000102430	ENSAMXG00000020921	–	–
	*opn6b*	ENSDARG00000098051	ENSAMXG00000008164	+	–
	*opn7a*	ENSDARG00000024208	ENSAMXG00000002437	–	–
	*opn7d*	ENSDARG00000068124	ENSAMXG00000013005	–	–
	*opn9*	ENSDARG00000104231	ENSAMXG00000018966	–	+
*rgr/rrh/opn5*	*opn5*	ENSDARG00000070110	XM_007239428.1 (NCBI)	–	–
	*rrh*	ENSDARG00000039534	ENSAMXG00000017584	–	+
	*rgra*	ENSDARG00000054890	ENSAMXG00000012172	+	+
	*rgrb*	ENSDARG00000098724	ENSAMXG00000004323	+	–
*Melanopsins*	*opn4m1*	ENSDARG00000022098	ENSAMXG00000021230	+	+
	*opn4m2*	ENSDARG00000007553	ENSAMXG00000025628	+	–
	*opn4m3*	ENSDARG00000053929	ENSAMXG00000001604	–	–
	*opn4x1*	ENSDARG00000079129	ENSAMXG00000006974	+	–
	*opn4x1*	ENSDARG00000079129	ENSAMXG00000006974	+	–

**Notes.**

Database search by querying gene sequences of the NCBI GenBank repository, Ensembl genebuild (Ensembl.org; AstMex 1.0.2, genebuild at July 2016) (McGaugh et al., 2014; Yates et al., 2016) (see ‘Materials and Methods’). +, positive hit in the database search; –, no hit in the database search within the whole adult tissue SRA database (Bioproject ID: PRJNA183542). *, *A. mexicanus* genome annotation has only one mws and no sws1 at Jan 2018.

The phylogenetic analysis including all zebrafish and *A. mexicanus* Opsins showed the same major opsin clades and topology as in Davies et al. (2015) (Fig. 1). For example, the Opsin super family has six major categories in vertebrates, all of which were represented by *A. mexicanus* and zebrafish orthologs. In addition, the phylogenetic distances between orthologs are closer than paralogs, which are consistent with those of zebrafish (e.g., Opn4m1, Opn4m2 and Opn4m3 in Fig. 1). Note that there is a difference between the positions of *A. mexicanus* Opsins and that of zebrafish (Davies et al., 2015): the melanopsin (Opn4) clade was placed as a sister to the Rgr/Rrh and Opn5/Opn6-9 clades, but in our analysis melanopsin was placed more basally, as a sister clade to all the other opsins included (Fig. 1). However, it is a relatively minor discrepancy, and overall most of the *A. mexicanus* Opsin sequences grouped with the zebrafish orthologs, suggesting that these *A. mexicanus* Opsin sequences are consistently annotated. It is noteworthy that we only detected one *mws* in both surface and cavefish instead of 4 *mws*, which were found in zebrafish (Table 2), and we could not detect *A. mexicanus sws1* in our search (Table 2). However, the loss or absence of *mws* and *sws* genes in the genome is difficult to conclude. The failure of detection may be due to an incomplete genome sequence that contains many unannotated regions. The ongoing genome sequencing project will help with investigating the loss of these genes.

Based on our BLASTn searches of the NCBI SRA database we concluded that 31 opsins (out of the 33 total recovered) were expressed in at least one embryonic/larval stage surveyed in either surface fish or cavefish (Table S6). The tissue transcriptome survey indicated that the surface fish eye expressed the largest number of *opsin* genes (26 genes), but surprisingly, a similar number of *opsins* (24) were expressed in extraocular tissues of cavefish (Table 1). Among these 24, the majority (20 *opsins*) were also expressed in the cavefish brain (Table 1). We therefore focused our subsequent comparative expression studies between surface fish and cavefish on eye and brain tissues.

### *Opsin* expression profile using RT-qPCR

We here first describe *opsin* gene expression in the eyes, followed by pineal and other brain tissues of surface fish and cavefish.

**Figure 3:**
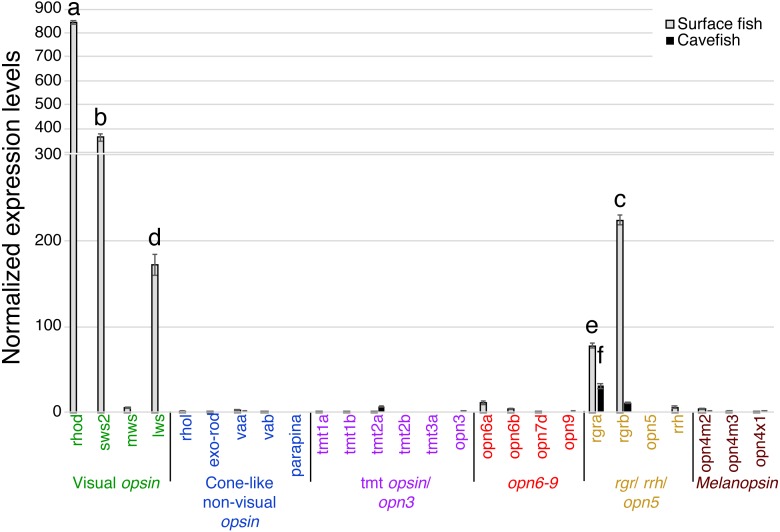
Normalized expression levels of 26 opsin genes in adult *A. mexicanus* eyes for surface fish and cavefish genes using RT-qPCR. Expression levels were calculated from Cq using the 2^−ΔΔ*Cq*^ method. Opsins are grouped into 6 clades: visual opsins (green), cone-like non-visual opsins (blue), *tmt* opsins/opn3 (purple), opn6-9 (red), *rgr/rrh/opn5* (orange), and melanopsins (black). Surface fish eyes expressed *rhodopsin*, *sws2*, *lws*, *rgra* and *rgrb*. In contrast, cavefish eyes expressed no opsin family genes at the comparable level of those of surface fish. Lowercase letters indicated the significant homogeneous subsets according to Turkey-b post-hoc test (alpha < 0.05). Unlabeled bars are not significantly different from zero.

In the binocular eyes (Fig. 3), normalized expression levels in 3 of the 4 visual opsins assayed were significantly higher in surface than cavefish, with *rhodopsin* (*rhod*) having the highest levels of expression, followed by the blue-sensitive *sws2* gene, and then the red-sensitive *lws* gene. The fourth visual opsin assayed, a green/yellow-sensitive *mws*, was expressed at low levels, close to the detection threshold in surface fish. In contrast, cavefish eyes expressed these 4 visual *opsins* at levels below the detection threshold. *rgra*, one of the teleost paralogs of *rgr* that is known to play a role in the retinoid cycle of the *trans* to *cis* conversion in vertebrates (i.e., photoisomerase), was expressed in detectable levels in both morphs. The surface fish eyes, however, showed significantly higher expression of *rgra* and *rgrb* than the degenerated eyes of the cavefish. In summary, as expected from former studies (Yokoyama et al., 1995; Hinaux et al., 2013; Gross et al., 2013; McGaugh et al., 2014), surface fish eyes expressed visual *opsins* at high levels whereas cavefish eyes expressed undetectable levels of visual *opsins*. However, it is new that cavefish eyes express *rgra* photoisomerase.

**Figure 4:**
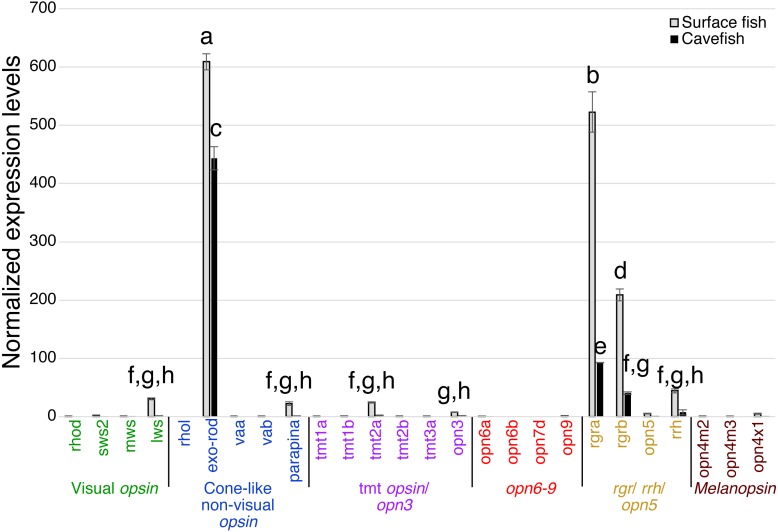
Normalized expression levels of 26 opsin genes in the adult *A. mexicanus* pineal for surface fish and cavefish using RT-qPCR. Expression levels were calculated from Cq using the 2^−ΔΔ*Cq*^ method. A post-hoc Tukey-b test was performed, separating significantly different means into groups labeled A–H. Cavefish pineal expressed a comparable, yet, significantly reduced expression level of the *exo-rod* gene. The *rgra* gene is also expressed at detectable levels in the cavefish pineal. However, in general, all opsin genes that are expressed in cavefish pineal are at significantly lower levels than those of surface fish. Lowercase letters indicated the significant homogeneous subset according to Turkey-b post-hoc correction (alpha < 0.05). Unlabeled bars are not significantly different from zero.

The pineal organ of teleosts is known as the central regulator of body-wide circadian rhythms by eliciting melatonin hormone during the dark period (Foulkes et al., 2016). In both surface fish and cavefish the pineal organ expressed high levels of the melatonin regulator *exo-rhodopsin* (*exo-rod*) (Pierce et al., 2008), in addition to *rgra* and *rgrb* (Fig. 4). It is noteworthy that cavefish *exo-rod* was expressed in the pineal at 72.7% of the expression levels in surface fish (443 vs 609 in normalized expression levels of cavefish and surface fish, respectively) (Fig. 4). Surface fish expression levels were also higher than in cavefish for *rgra* and *rgrb*. Gene expression was also detectable in surface fish pineal for *lws, parapinopsin-a* (*parapina*) that is expressed in pineal and para-pineal in zebrafish and is suggested to contribute to photic regulation of circadian rhythms (Shichida & Matsuyama, 2009)*,* a blue-light sensitive *teleost multiple tissue opsin 2a* (*tmt2a*) whose proposed function is to regulate photic entrainment of peripheral clocks (Moutsaki et al., 2003)*,* and a blue-light sensitive *opsin 3* (*opn3* or *encephalopsin*) (Sakai et al., 2015). The pineal of both morphs also expressed *retinal pigment epithelium-derived rhodopsin homolog* (*rrh*) whose suggested role is a photoisomerase similar to *rgr* (Bellingham, Wells & Foster, 2003). In summary, the functions of most of the Opsins expressed in the pineal were related to photic entrainment-regulation, i.e., light-dependent regulation of the circadian rhythm, rather than visual-regulation.

**Figure 5:**
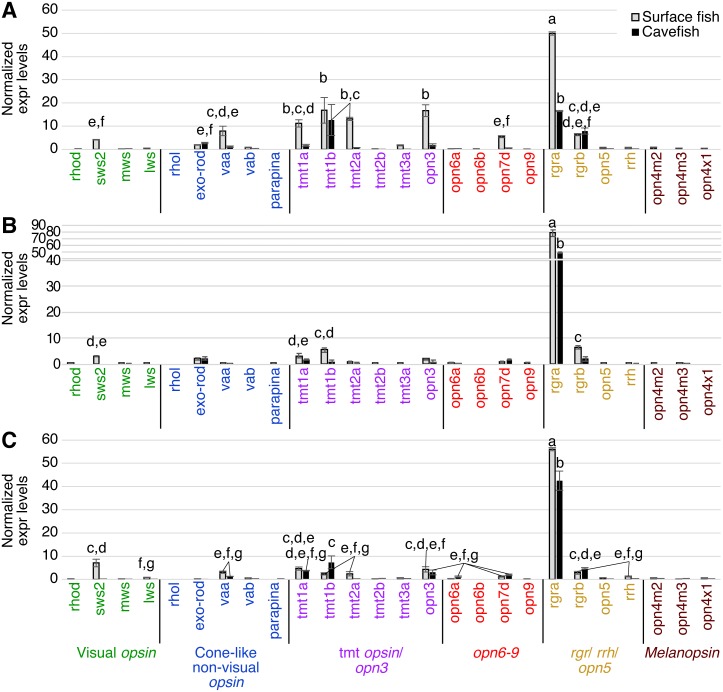
Normalized expression levels of 26 opsin genes in three adult *A. mexicanus* brain tissues for surface fish and Pachon cavefish using RT-qPCR. (A) Tectum; (B) Telencephalon; (C) Midbrain and hindbrain. Expression levels were calculated based on Cq using the 2^−ΔΔ*Cq*^ method. Tectum (A) showed multiple opsin expression including *sws2*, *exo-rod, tmt1a, tmt1b tmt2a, opn3, opn7d, rgra,* and *rgrb* in surface fish. Expression level of the majority of these opsin genes were attenuated in cavefish, yet some of the gene are expressed at detectable levels such as *tmt1b, rgra,* and *rgrb*. Lowercase letters indicated the significant homogeneous subset according to Turkey-b post-hoc correction (alpha < 0.05). Unlabeled bars are not significantly different from zero.

The tectum expressed a variety of photoisomerase- and photoreceptor-type opsins in both surface and cavefish (Fig. 5A). A potential light-sensing ability for the tectum was previously suggested based on the expression of *tmt* and *va* opsins, and its light-dependent electrophysiological response was reported in adult medaka (*Oryzias latipes*) (Fischer et al., 2013). In *A. mexicanus*, the highest expression detected was from surface fish *rgra*. Interestingly, cavefish *rgra* was also highly expressed. It is also noteworthy that *tmt1b* was significantly expressed in both surface- and cavefish morphs at similar levels. Photoisomerase-type *rgrb* opsin was also expressed at similar levels in both morphs. In addition, the surface fish tectum expressed *opn3, tmt1a, tmt2a, opsin 7d* (*opn7d*), low level of *sws2,* and low level of *vertebrate ancient opsin a* (*vaa*), which was first identified in horizontal cells of the retina and works as a photosensing pigment (Jenkins et al., 2003). These genes were not detected in the cavefish tectum. It is also interesting that cavefish *exo-rod*, whose known function is to regulate melatonin at the pineal organ, was expressed at a detectable level in the tectum.

In the telencephalon, for which a photosensing ability has not previously been reported, the highest expression levels found for an opsin, for both surface and cavefish, was for *rgra* (Fig. 5B). Expression of surface fish *rgrb, tmt1b, tmt1a* and *sws2* were also detected, although at very low levels. Other than *rgra,* the cavefish telencephalon did not express any opsins above the detection threshold.

In deep brain tissue, the opsin gene with the highest expression, in both surface fish and cavefish, was again *rgra*, although the level of the cavefish *rgra* expression was significantly lower than that of surface fish (Fig. 5C). It is important to note that many other opsins were also expressed in the deep brain, revealing that the deep brain has a greater suite of opsins expressed than in the eyes of either morph (Figs. 3–5). For example, the deep brain of both cave and surface morphs expressed *rgrb, vaa, tmt1a, tmt1b, opn3,* and *opn7d*. The surface fish deep-brain additionally expressed visual opsins—*sws2* and *lws*—and *tmt2a* and *rrh* opsins. It is interesting that the cavefish deep brain expressed *opn6a*, another novel Opsin class (Davies et al., 2015), which was not observed in surface fish. It is worth noting that Melanopsin (Opn4) has been linked to the regulation of negative phototaxis in zebrafish larvae (Fernandes et al., 2012). However, we did not detect *melanopsin* expression (*opn4*) in the deep brain tissue of adult *A. mexicanus*.

### Diurnal activity pattern is maintained after removal of the pineal

**Figure 6:**
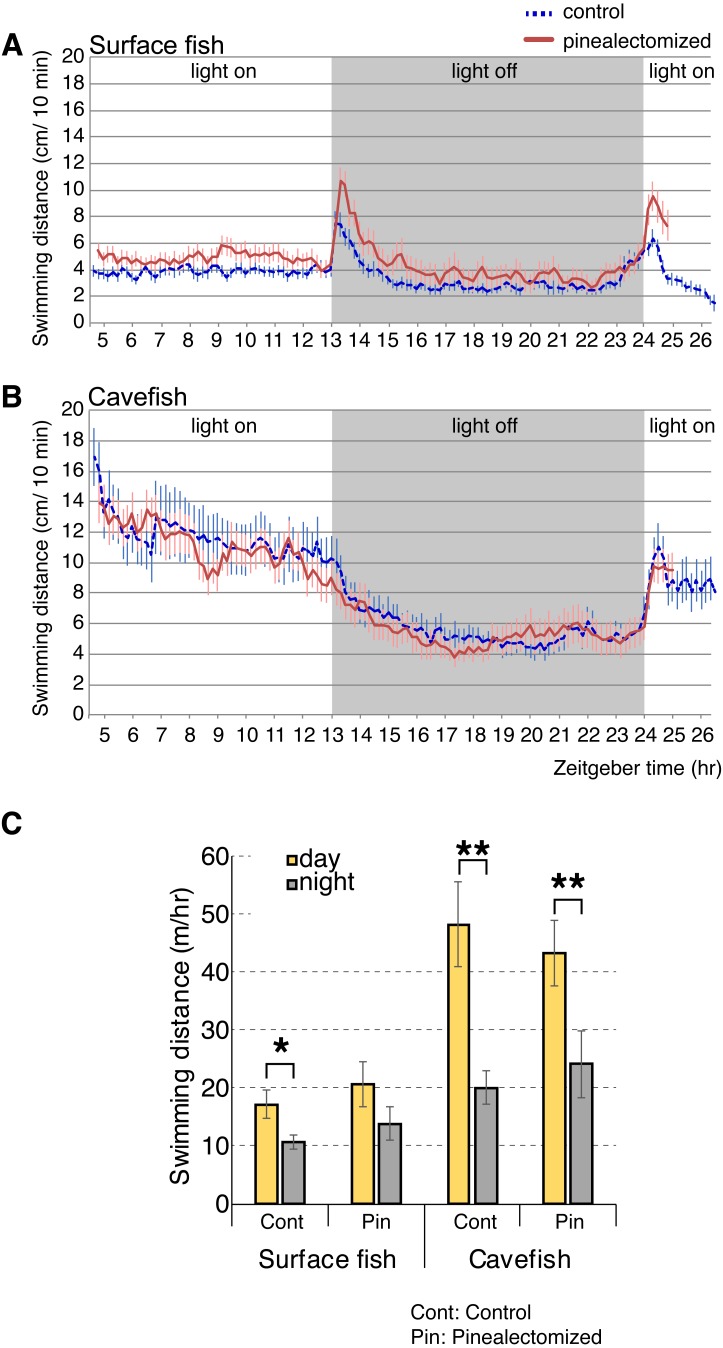
Activity shift of juvenile surface fish and cavefish according to the light/dark environment. (A) Surface fish actogram during light and dark condition. *X*-axis indicate Zeitgeber time (ZT) and *Y*-axis indicate average distance fish swim within 10 min bin. Vertical bars represent standard error of means. Lines indicate the average of fish swimming distance in each 10 min. (B) Cavefish actogram during light and dark condition. (C) Summary of activity change during light and dark condition. Statistics are available in Tables S7 and S8. Stars indicate student’s *t*-test results after Bonferroni correction, *, *P* < 0.05; **, *P* < 0.01.

According to our analyses of opsin gene expression, the cavefish pineal tissue expressed the highest levels of light-sensing opsins (i.e., non-photoisomerase opsins) among the eye and brain tissues tested. The pineal cells expressed the melatonin-regulating *exo-rod*, which may have another role in addition to regulating the level of plasma melatonin in *A. mexicanus*. For example, *A. mexicanus* pineal is known to regulate shadow responses where larval fish swim upward in response to a shadow (Yoshizawa & Jeffery, 2008). Therefore, we hypothesized that the *exo-rod*-rich pineal of cavefish regulates the light-dependent basal activity. We first performed pinealectomies in early stage larvae, and then raised them to the juvenile stage (∼16 weeks old). We then tracked the activity levels of surface fish and cavefish in a 24 h period. Both pinealectomized and control surface fish showed higher activity in the light and lower activity in the dark (Fig. 6A). It is worth mentioning that, from the actogram (Fig. 6A) pinealectomized surface fish showed a 10 min delay in response to the ‘dark’ stimulus when compared with the control. In cavefish, as in surface fish, pinealectomized and control fish also showed higher activity in the light and lower activity in the dark (Fig. 6B). When we compared the day and night activities in these fish (Fig. 6C), our hypothesis—that the cavefish pineal may regulate the activity shift between day and night—failed to be supported because both the surgery control and pinealectomized cavefish showed similar levels of significant diurnal change in their activities (between day and night: *N* = 18, *t*_17_ = 3.9, *P* < 0.01; and *N* = 15, *t*_14_ = 3.8, *P* < 0.01 after Bonferroni correction, for control and pinealectomized cavefish, respectively Tables S7 and S8). In contrast, in surface fish, in which it is presumed that the pineal regulates the circadian clock as in other teleosts (Foulkes et al., 2016), the day-night activity change was undetectable in the pinealectomized fish (between day and night: *N* = 15, *t*_14_ = 2.1, *P* > 0.05, after Bonferroni correction; Figure 6C and Tables S7 and S8), whilst the control surface fish showed a significant shift of locomotor activity between day and night (*N* = 18, *t*_17_ = 3.0, *P* < 0.028, after Bonferroni correction). Similar to previous studies (Duboué, Keene & Borowsky, 2011; Moran, Softley & Warrant, 2014; Yoshizawa et al., 2015), both control surface and cavefish showed diurnal patterns of activities (Figs. 6A–6C). It is also important to note that both control and pinealectomized surface fish showed a large burst in swimming following both the light OFF and ON (Fig. 6A). This phenomenon is also observed in zebrafish larvae (the so-called “masking”), and was suggested as an escape response from the dark and light-elevated response (Burgess & Granato, 2007b). This light-dependent swimming burst was not observed in both the control and pinealectomized cavefish in the transition from light to dark (Fig. 6B). However, there was a small light-dependent burst in response to the transition from dark to light, with no observed differences in response between control and pinealectomized cavefish (Fig. 6B). The existence of a light-dependent response suggests that this cavefish burst behavior is regulated by a non-pineal organ. As reported, a diurnal activity shift was observed in control cavefish (*P* = 0.005), and importantly, in pinealectomized cavefish as well (*P* = 0.008). These results suggest that the pineal gland contributes little to the regulation of light-dependent basal activity in either surface fish or cavefish.

## Discussion

We identified a conservative set of 33 opsin gene fragments in the *A. mexicanus* genome. Analysis of the transcriptomes from eye and brain tissues revealed that a large proportion of these opsins were expressed at detectable levels in both surface and cavefish. As the majority of opsins were expressed in eyes and brain, we focused primarily on these tissues and performed RT-qPCR to compare the expression levels of 26 opsins in the eyes, pineal, optic tectum, telencephalon and deep brain in surface and cave morphs. Our result suggested that, despite 2,000–20,000 years of cave dwelling, some non-visual *opsins* are still highly expressed in the brain tissues of cavefish.

### exo-rod expression and involvement of the pineal in the light-dependent locomotor activity

According to our study, many opsin genes for vision and light-detection pathways (*rhod, sws2, lws, tmt, va,* and *opn3*) were severely downregulated in cavefish tissues as compared with surface fish. This phenomenon was obvious in the visual opsins expressed in the eyes, which is likely due to the significant reduction of photoreceptor cells (Wilkens, 1988; Langecker, Schmale & Wilkens, 1993; Yamamoto & Jeffery, 2000). In contrast, photoisomerase opsins (*rgr* and *rrh*) in cavefish were expressed at comparable levels to surface fish in many tissues. Among these opsins, the light-sensing *exo-rhodopsin* is unique because it is involved in regulating melatonin according to ambient light, and the expression level of *exo-rod* in the cavefish pineal was still high relative to its expression in surface fish (443 in cavefish, 609 in surface fish in normalized expression levels). The slight down regulation of *exo-rhodopsin* in the cavefish pineal can be explained by a reported fact that the outer segments of photosensory cells are not well developed in a cave population (Chica population; we used Pachón cave population) (Wilkens, 1988). Previous studies have characterized *exo-rod* as a pineal-specific opsin in teleosts (Mano, Kojima & Fukada, 1999). The expression pattern of *A. mexicanus exo-rod* in surface fish and cavefish, reported here, is comparable to zebrafish, which have high levels of expression in the pineal and low levels in the eyes and other brain regions (Davies et al., 2015). In zebrafish, *exo-rod* has been shown to be involved in the synthesis of melatonin (Pierce et al., 2008). Knockdown of *exo-rod* using antisense morpholinos in zebrafish resulted in lowered expression of *aanat2*, which codes for a key enzyme in melatonin synthesis (Pierce et al., 2008). Melatonin levels affect many aspects of an organism’s physiology, including locomotor activity levels (Falcón et al., 2010). Therefore, we hypothesized that photoreception in the pineal and downstream effects on melatonin levels may be involved in the light-induced shift in activity levels observed in *A. mexicanus* cavefish. Surprisingly, our study did not detect a role for the pineal in the regulation of light-dependent locomotor activity in either surface fish nor cavefish. However, an additional factor needs to be considered in this study: the relatively small size of the arenas for juvenile fish (a well-arena with a 3.48 cm diameter, compared with an ∼1.5cm length test fish). This may make fish nervous and induce unexpected responses, although we detected both the day and night rhythm and the light-dependent swimming burst in surface fish similar to the one described in zebrafish (Burgess & Granato, 2007b). Therefore, at this moment, there is a need for future studies to address whether space constraints affect *Astyanax* behavior in the light and the dark, and/or the exhibition of swimming bursts. Nonetheless, it is likely that exo-rod and pineal are not involved in the light-induced shift in basal activity of cavefish.

### Green-sensing opsin in surface fish

An unexpected result from our expression data was the low level of expression of the *mws* visual opsin in surface *Astyanax* because *mws* is one of the major visual opsins expressed in vertebrate eyes. This gene was expressed at low levels in all tissues assayed, including the eye. This raises two possibilities: (1) our primer sets for *mws* were not efficient in amplifying the target, and/or (2) the low expression level is genuine. The *mws* used here is related to the zebrafish ‘rh2-group’ *mws* genes. We did not include the other, related medium-wavelength-sensitive opsins *g101* or *g103* in our RT-qPCR assays, but it is possible that one or both of these function as green-sensitive visual opsins in surface fish instead of the *A. mexicanus mws.* Further investigation is needed to resolve this issue.

### Evolution of the light-sensitive opsin genes in the cave-ecosystem

Cave dwelling *A. mexicanus* have evolved in perpetual darkness for 20,000 years or more (Mitchell, Russell & Elliott, 1977; Ornelas-García, Domínguez-Domínguez & Doadrio, 2008; Fumey et al., 2018). During this period, the ability to detect light may have been deleterious given that binocular eyes show high energy consumption (Moran, Softley & Warrant, 2015). Our study supports previous finding that the expressions of vision/eye-related genes including visual opsins are severely attenuated in the dark adapted cave morph of *A. mexicanus* (Yokoyama et al., 1995; Hinaux et al., 2013; Gross et al., 2013; McGaugh et al., 2014). However, pineal opsin (Exo-rod) and other non-visual opsins including *tmt, va, rgr* and *rrh* are still expressed in the cavefish brain (Figs. 4 and 5).

The *exo-rod* expressing pineal of both cavefish and surface fish did not significantly regulate light dependent locomotor activity, suggesting the major role of other, not yet identified, tissue(s) and opsin(s) for the regulation of this light-dependent behavior. Exo-rod is involved in the synthesis of melatonin in zebrafish in a light-dependent manner (Pierce et al., 2008). There is currently no clue as to why the *exo-rod* expression has been preserved in the cavefish pineal. One possibility is that the pineal does not consume as much resting-energy as the large binocular eyes in the dark (cf. Moran, Softley & Warrant, 2015). In other words, the *exo-rod* expression might be an evolutionary relict because it is non-deleterious in the cave environment, or might have an as yet unknown function in the pineal. Selection pressure analyses, such as divergent (*F*_ST_, *D*_*XY*_), heterozygosity and/or haplotype calculations, for the flanking genomic sequence of the *exo-rod* locus may reveal whether the *exo-rod* locus is actively conserved or not.

### Evolution of Opsins involved in retinal metabolism

Photoisomerase RGR opsins were significantly expressed in eyes, the pineal, tectum and deep brain of cavefish (Figs. 3–5). RGR opsins were originally characterized in the retinal pigment epithelium (RPE) of mammals (Jiang, Pandey & Fong, 1993). Our findings of *rgr* expression in the pineal and other brain regions of both surface and cavefish are in agreement with reported expression data in zebrafish. Davies et al. (2015) showed high expression in the eye and pineal for *rgr1* and *rgr2* (homologous to *Astyanax rgra* and *rgrb*, respectively). Further, the most highly expressed opsin in the zebrafish brain was *rgr1*, with lower levels detected for *rgr2*, which is corroborated by the data reported here for *A. mexicanus*. The expression of cavefish *rgr* opsins suggests two possibilities: (i) *rgr* expression is not deleterious so that it remains as an evolutionary residual, or (ii) *rgr* opsins have an unknown physiological function that has been retained in the absence of light. It is noteworthy that Carlson et al. (2018) showed that *rgra* was upregulated 2-2.5 fold in cavefish embryos compared with surface fish at the same stage (10-24 h post fertilization). However, there were no detectable differences in *rgra* expression between cave and surface fish in this study. These results may suggest the possibility that Rgr plays an unknown developmental role such as inducing light dependent DNA repair genes (*CPD phr*) in cavefish embryos of *A. mexicanus* (Frøland Steindal et al., 2018), and/or may regulate light-dependent activity in both surface fish and cavefish in the same way. Future studies could test these possibilities by using genome editing to knock-out *rgr* expression and then assay the resulting developmental/physiological phenotypes.

### Swimming burst in response to light-on and light-off

There is a non-directional (non-phototaxis) swimming burst in surface fish following light-ON and OFF stimuli (Fig. 5A). This behavioral response to a change in lighting condition is similar to a known larval behavior in zebrafish (Burgess & Granato, 2007a). However, it is important to note that the adaptation time, which is a duration between when the burst started and when the activity returned at the normal level, to the light/dark condition in the current study was longer than the zebrafish study (∼2 h versus ∼30 min in *A. mexicanus* surface fish juvenile and zebrafish larvae, respectively) (Burgess & Granato, 2007a; Burgess & Granato, 2007b). It is also noteworthy that just before the light ON, both control and pinealectomized surface fish seemed to increase their activity levels (Fig. 6A), suggesting that swimming burst—especially at light ON—could also be regulated by circadian rhythm without light input*.* This discrepancy may be due to species differences between *A. mexicanus* and zebrafish, and/or difference in the developmental stage (larvae versus juveniles).

A previous study has shown that some interneurons in the tectum of zebrafish and medaka fish express *tmt* and *va* opsins and are able to respond to light (Fischer et al., 2013). In the *A. mexicanus* tectum we detected the expression of multiple *tmt opsins*, as well as *sws2, exo-rod, vaa, opn3, opn7d* and both *rgr* genes. This result suggests a possible novel function of the tectum in which tectum neurons may adjust the visual information from binocular eyes by sensing ambient light, in addition to a possibility to regulate the light-dependent basal activity. Such exciting possibilities could be tested in genetically modified *A. mexicanus* as described above via genome editing.

### Opsin regulation and circadian rhythm

In vertebrate species, some *opsin* expression is under the regulation of circadian rhythms (Li & Dowling, 1998; McMahon, Iuvone & Tosini, 2014). In this study, we have dissected fish between ZT2-ZT5 and did not recover tissues at other time points. It would be interesting to study *opsin* gene regulation at different circadian times to address whether *A. mexicanus opsin* expression levels are under circadian regulation.

## Conclusions

In summary, we here reported 33 *opsins* from the *A. mexicanus* genome, many of which are expressed at detectable levels in multiple surface and cavefish tissues. All of the brain tissues of cavefish investigated here retained the expression of photoisomerase opsins (*rgr*) and non-visual opsin *tmt*, suggesting a possibility that these opsins may have retained an unknown physiological function in the dark. Also, surprisingly, the *exo-rod* was expressed by the pineal gland of both surface fish and cavefish but is revealed to have little contribution to light-dependent locomotor activity. This initial survey will serve as a foundation to investigate the evolutionary regression of opsin genes under darkness, and the potential for light-independent roles of opsins.

##  Supplemental Information

10.7717/peerj.8148/supp-1 Supplemental Information 1 Supplemental Tables

10.7717/peerj.8148/supp-2 Supplemental Information 2 Raw qPCR data for the expression of 25 genes The expression of 25 genes whose expression level was standardized by the expression of three housekeeping genes *b2m*, *eef2a.1* and *rps18.* Cf: cavefish, Sf: surface fish, tel: telencephalon.

10.7717/peerj.8148/supp-3 Supplemental Information 3 Raw data for waking activity in pinealectomized fish Waking activity (m per 10 min) in pinealectomize surface fish (Sf) and cavefish (Cf). The data of daytime (5hr:ZT5-9) and night time (5 h: ZT18-21) were used to analyze the activity to avoid the influence of masking (see body text).

## Additional Information and Declarations

### Data Availability

The following information was supplied regarding data availability: Yoshizawa, Masato, Simon, Noah, Fujita, Suguru, & Porter, Megan. (2019). Expression of Extraocular opsin Genes and Light-Dependent Basal Activity of Blind Cavefish. Zenodo. http://doi.org/10.5281/zenodo.3533322.
