# Supplementary material for: Expression of extraocular opsin genes and light-dependent basal activity of blind cavefish
Source: PeerJ. 2019 Dec 17;7:e8148. doi: 10.7717/peerj.8148 (PMC6924323; doi:10.7717/peerj.8148)
Supplement: Supplemental Information 1 [file peerj-07-8148-s001.docx]

**SUPPLEMENTARY MATERIALS**

**Table S1. Accession numbers for reference genes used and opsin genes found for *A. mexicanus* and zebrafish opsins used to search for homologs.**

| **Gene name** | ***A.mexicanus* accession #** | **Zebrafish accession #** |
| --- | --- | --- |
| *b2m* | ENSAMXG00000011344 |  |
| *eef2a.2* | ENSAMXG00000018020 |  |
| *rps18* | ENSAMXG00000007922 |  |
| *rhod* | ENSAMXG00000026346 | ENSDARG00000002193 |
| *sws2* | ENSAMXG00000006363 | ENSDARG00000017274 |
| *mws* | ENSAMXG00000001266 | KT008395 (NCBI) |
| *lws1* | ENSAMXG00000006368 | ENSDARG00000044862 |
| *lws2* | (No expression) | ENSDARG00000044861 |
| *rhol* | ENSAMXG00000024894 | KT008393 (NCBI) |
| *exo-rod* | ENSAMXG00000017182 | ENSDARG00000103574 |
| *vaa* | ENSAMXG00000009826 | ENSDARG00000054181 |
| *vab* | ENSAMXG00000010897 | ENSDARG00000054181 |
| *tmt1a* | ENSAMXT00000017410 | ENSDARG00000103674 |
| *tmt1b* | ENSAMXG00000008135 | ENSDARG00000032246 |
| *tmt2a* | ENSAMXG00000009550 | KT008407 (NCBI) |
| *tmt2b* | ENSAMXG00000003866 | ENSDARG00000027822 |
| *tmt3a* | ENSAMXG00000019922 | ENSDARG00000036460 |
| *opn3* | ENSAMXG00000020951 | ENSDARG00000052775 |
| *opn6a* | ENSAMXG00000020921 | ENSDARG00000102430 |
| *opn6b* | ENSAMXG00000008164 | ENSDARG00000098051 |
| *opn7a* | ENSAMXG00000002437 | ENSDARG00000024208 |
| *opn7d* | ENSAMXG00000013005 | ENSDARG00000068124 |
| *opn9* | ENSAMXG00000018966 | ENSDARG00000104231 |
| *rgra* | ENSAMXG00000012172 | ENSDARG00000054890 |
| *rgrb* | ENSAMXG00000004323 | ENSDARG00000098724 |
| *opn5* | ENSAMXG00000010179 | ENSDARG00000070110 |
| *rrh* | ENSAMXG00000017584 | ENSDARG00000039534 |
| *opn4m2* | ENSAMXG00000025628 | ENSDARG00000007553 |
| *opn4m3* | ENSAMXG00000001604 | ENSDARG00000053929 |
| *opn4x1* | ENSAMXG00000006974 | ENSDARG00000079129 |
| *g101* | ENSAMXG00000001701 | N/A |
| *g103* | ENSAMXG00000001722 | N/A |
| *parapinopsina* | ENSAMXG00000014941 | KT008404 |
| *parapinopsinb* | ENSAMXG00000007169 | ENSDARG00000044672 |
| *parietopsin* | ENSAMXG00000010213 | KT008406 |
| *opn4m1* | ENSAMXG00000021230 | ENSDARG00000022098 |

**Table S2. Amino acid accession numbers used for phylogenetic analysis.**

| **Protein name** | **Accession #** |
| --- | --- |
| Cavefish vab | ENSAMXP00000011222 |
| Cavefish vaa | ENSAMXP00000010101 |
| Cavefish tmt3a | ENSAMXP00000020512 |
| Cavefish tmt2b | ENSAMXP00000003950 |
| Cavefish tmt2a | ENSAMXP00000009817 |
| Cavefish tmt1b | ENSAMXP00000008357 |
| Cavefish tmt1a | ENSAMXP00000017410 |
| Cavefish sws2 | ENSAMXP00000006531 |
| Cavefish rrh | ENSAMXP00000018103 |
| Cavefish rhol | ENSAMXP00000025600 |
| Cavefish rhod | ENSAMXP00000027051 |
| Cavefish rgrb | ENSAMXP00000004427 |
| Cavefish rgra | ENSAMXP00000012519 |
| Cavefish parietopsin | ENSAMXP00000010487 |
| Cavefish parapinb | ENSAMXP00000007364 |
| Cavefish parapina | ENSAMXP00000015377 |
| Cavefish opn9 | ENSAMXP00000019534 |
| Cavefish opn7d | ENSAMXP00000013374 |
| Cavefish opn7a | ENSAMXP00000002485 |
| Cavefish opn6b Ensembl | ENSAMXP00000008387 |
| Cavefish opn6b NCBI | XP_022528332.1 (NCBI) |
| Cavefish opn6a | ENSAMXP00000021560 |
| Cavefish opn5 | ENSAMXP00000010451 |
| Cavefish opn4x1 | ENSAMXP00000007163 |
| Cavefish opn4m3 | ENSAMXP00000001657 |
| Cavefish opn4m2 | ENSAMXP00000026333 |
| Cavefish opn4m1 | ENSAMXP00000021860 |
| Cavefish opn3 | ENSAMXP00000021567 |
| Cavefish mws | ENSAMXP00000001292 |
| Cavefish lws1 | ENSAMXP00000006533 |
| Cavefish g103 | ENSAMXP00000001750 |
| Cavefish g101 | ENSAMXP00000001733 |
| Cavefish exo-rod splice variant 2 | ENSAMXP00000017689 |
| Cavefish exo-rod splice variant 1 | ENSAMXP00000017687 |
| Zebrafish exo-rod | ALG92531 (NCBI) |
| Zebrafish rhod | ALG92532 (NCBI) |
| Zebrafish rhol | ALG92533 (NCBI) |
| Zebrafish mws1 | ALG92534 (NCBI) |
| Zebrafish mws2 | ALG92535 (NCBI) |
| Zebrafish mws3 | ALG92536 (NCBI) |
| Zebrafish mws4 | ALG92537 (NCBI) |
| Zebrafish sws2 | ALG92538(NCBI) |
| Zebrafish sws1 | ALG92539 (NCBI) |
| Zebrafish lws1 | ALG92540 (NCBI) |
| Zebrafish lws2 | ALG92541 (NCBI) |
| Zebrafish vaa | ALG92542 (NCBI) |
| Zebrafish vab | ALG92543 (NCBI) |
| Zebrafish parapinopsina | ALG92544 (NCBI) |
| Zebrafish parapinopsinb | ALG92545 (NCBI) |
| Zebrafish parietopsin | ALG92546 (NCBI) |
| Zebrafish tmt1a | ALG92547 (NCBI) |
| Zebrafish tmt1b | ALG92548 (NCBI) |
| Zebrafish tmt2a | ALG92549 (NCBI) |
| Zebrafish tmt2b | ALG92550 (NCBI) |
| Zebrafish tmt3a | ALG92551 (NCBI) |
| Zebrafish tmt3b | ALG92552 (NCBI) |
| Zebrafish opn3 | ALG92553 (NCBI) |
| Zebrafish opn5 | ALG92554 (NCBI) |
| Zebrafish opn6a | ALG92555 (NCBI) |
| Zebrafish opn6b | ALG92556 (NCBI) |
| Zebrafish opn7a | ALG92557 (NCBI) |
| Zebrafish opn7b | ALG92558 (NCBI) |
| Zebrafish opn7c | ALG92559 (NCBI) |
| Zebrafish opn7d | ALG92560 (NCBI) |
| Zebrafish opn8a | ALG92561 (NCBI) |
| Zebrafish opn8b | ALG92562 (NCBI) |
| Zebrafish opn8c | ALG92563 (NCBI) |
| Zebrafish opn9 | ALG92564 (NCBI) |
| Zebrafish rrh | ALG92565 (NCBI) |
| Zebrafish rgra | ALG92566 (NCBI) |
| Zebrafish rgrb | ALG92567 (NCBI) |
| Zebrafish opn4m1 | ALG92568 (NCBI) |
| Zebrafish opn4m2 | ALG92569 (NCBI) |
| Zebrafish opn4m3 | ALG92570 (NCBI) |
| Zebrafish opn4x1 | ALG92571 (NCBI) |
| Zebrafish opn4x2 | ALG92572 (NCBI) |
| Bovine rhod | NP_001014890 (NCBI) |
| *Homo sapiens* melatonin receptor type 1A | NP_005949.1 (NCBI) |
| *Homo sapiens* melatonin receptor type 1B | NP_005950.1 (NCBI) |
| *Homo sapiens* probable G-protein coupled receptor 21 | NP_005285.1 (NCBI) |
| *Homo sapiens* probable G-protein coupled receptor 52 | NP_005675.3 (NCBI) |
| *Trichoplax adhaerens* hypothetical protein TRIADDRAFT_26009 | XP_002112437.1 (NCBI) |
| *Trichoplax adhaerens* hypothetical protein TRIADDRAFT_58712 | XP_002114830.1 (NCBI) |

**Table S3. Primer sequences for *A. mexicanus* reference and target genes used in RT-qPCR.**

| Gene name | Ensembl gene ID | Forward primer sequence | Reverse primer sequence | Efficiency (%) |
| --- | --- | --- | --- | --- |
| *b2m* | ENSAMXG00000018020 | TTCACACCTCAGAAGAACGA | ACTGCATTCTCCATCTGGT | 93.7 |
| *eef2a.2* | ENSAMXG00000018020 | TATCATTGAGGAGTCTGGAGAG | TGGGTCGGATTTCTTAATTGG | 100.5 |
| *rps18* | ENSAMXG00000007922 | CCATCAAGGGTGTTGGTAGG | TGCATAATGGTCACCACCC | 99.5 |
| *rhod* | ENSAMXG00000026346 | TGTGGCTGACTTGTTCATGG | GCACCAGAGTGAGTTGATACC | 112.2 |
| *sws2* | ENSAMXG00000006363 | AACAAACAGTTCCGCTCC | GGTCCTACAGAAGACACCT | 107.8 |
| *mws* | ENSAMXG00000001266 | CAACACTTGGAGGTGAAGTGG | AGGTCATTGCCATGAACCAG | 117.3 * |
| *lws* | ENSAMXG00000006368 | TATTGGCCAGCACCATCAG | CAGCAATACCACAGGTAGCA | 91.3 |
| *rhol* | ENSAMXG00000024894 | CTCAACGCTCTACAACCCT | GGCTTCTCAAACGGATTCTC | 179.4 * |
| *exo-rhod* | ENSAMXG00000017182 | CTCATGGTCACCTCATTTCCT | TGTGCGGAGCTTCTTGTG | 134.3 * |
| *vaa* | ENSAMXG00000009826 | GAATAAACAGTTCAGGAAGTGC | TGTGGTCTGGTTCACCTC | 103.9 |
| *vab* | ENSAMXG00000010897 | CCAAACTGGTACTCAACAGAC | GCATGAGTTTAGCATAAGAGCC | 116.1 * |
| *parapina* | ENSAMXG00000014941 | CATGAACAGACAGTTCAGGGA | GGAGACGATGGTGAAACCT | 0 * |
| *tmt1a* | ENSAMXT00000017410 | GCAATCAAACAGGTCAGTGGA | AGCAGAGCAGGTAGCAGAC | 88.9 * |
| *tmt1b* | ENSAMXG00000008135 | CATCAAGCAGGTGAGCAG | CCAGCACAACAGGTAACAC | 91.4 * |
| *tmt2a* | ENSAMXG00000009550 | TGAACAAACAGTTCTACAGGTG | GGATGACCGTAGTTCTGGA | 94.1 * |
| *tmt2b* | ENSAMXG00000003866 | TCCTGCTTCGGTATCGTATCTC | GGACCCTTCTTGTTGTACACC | 339.2 * |
| *tmt3a* | ENSAMXG00000019922 | CTTCAACAACCAGTTCTACAGG | GGGTTCAGGGTTTGTATAGAGG | 83.4 * |
| *opn3* | ENSAMXG00000020951 | TCGCCATTATCCCGTCCT | CAACGACGAAACTTTCTGCTC | 113.5 * |
| *opn6a* | ENSAMXG00000020921 | ACCCGAACAAAGCACACTG | ACCCTCGATCTGTGTAACTTCC | 94.7 |
| *opn6b* | ENSAMXG00000008164 | ATCACATCAACAAGCGTAAGGT | ATATCTGCGGGCTCTATAGCTG | 115.1 |
| *opn7a* | ENSAMXG00000002437 | ATTATAGTGAAGCTCAGTGTGG | TGGAATGACCTCTACATCCC | N/A ** |
| *opn7d* | ENSAMXG00000013005 | ATTGTGAACTTGTCCATCAGCG | AAACAGCCACTTGTGAGCG | 144.2 * |
| *opn9* | ENSAMXG00000018966 | AATGTCAAGCCTGTTTCATGGT | CAGCGGTCCAGAGAAATGAG | 318.4 * |
| *rgra* | ENSAMXG00000012172 | TGTTATGTCCTGCTACAAATCC | GTGCTACAGCTGAACTTATCC | 108.9 |
| *rgrb* | ENSAMXG00000004323 | GATCCGAATGGTTCTTCCAG | CCTCTTGTCTGTCTGGCT | 103 |
| *opn5* | ENSAMXG00000010179 | GCGGAGTCTGGATTATGGAG | GTCTGCTTCTTGTCTAGGAGTG | 255.4 * |
| *rrh* | ENSAMXG00000017584 | CATCGTCGCTGGATACCT | CCAGGTTAATGATGATAGCGT | 156.3 * |
| *opn4m2* | ENSAMXG00000025628 | ATGAAGAGTGAATGGAAGATGG | TGTAAGGAGTGAGGTGATGG | 116.5 |
| *opn4m3* | ENSAMXG00000001604 | GCAAGAGTCGAAGTCTGAG | TACAACTCACAACCTTTCTCC | 113.6 * |
| *opn4x1* | ENSAMXG00000006974 | CATCTCCATTTCCAACAGCGA | CATCACTCCAGACTGTATCACC | 195.2 * |

* Low expression in the efficiency assay gave limited data points to generate the standard curve

** Melt curve analysis indicated the primer set had multiple pcr products

**Table S4. Cycle specifications used in RT-qPCR assays.**

| RT-qPCR step | | Temp. (°C) | Time (s) |
| --- | --- | --- | --- |
| Polymerase activation /  DNA denaturation | | 95 | 30 |
| Denaturation | 40 cycles | 98 | 5 |
| Annealing/Extension  + Plate read |  | 60 | 30 |
| Melt curve analysis | 60 steps | 65-95 (increments of 0.5) | 5 (each step) |

**Table S5. Homologs of Zebrafish *opsins* (Davies et al., 2015) in *A. mexicanus* Databases and Expressions in surface and cavefish.**

|  | Gene name | Zebrafish Gene ID | *A. mexicanus* Gene ID | Surface fish | Cavefish |
| --- | --- | --- | --- | --- | --- |
| Visual *opsins* | *rhod* | ENSDARG00000002193 | U12328.1 (NCBI) | - | - |
|  |  |  | ENSAMXG00000026346 | + | - |
|  |  |  | Transcriptome | + | + |
|  | *rhol* | KT008393 (NCBI) | ENSAMXG00000024894 | - | - |
|  | *mws1* | KT008394 (NCBI) | ENSAMXG00000001266* | - | - |
|  | *mws2* | KT008395 (NCBI) |  |  |  |
|  | *mws3* | KT008396 (NCBI) |  |  |  |
|  | *mws4* | KT008397 (NCBI) |  |  |  |
|  | *sws1* | KT008398 (NCBI) | N/A* | N/A | N/A |
|  | *sws2* | ENSDARG00000017274 | AH007939.1 (NCBI) | + | + |
|  |  |  | ENSAMXG00000006363 | - | - |
|  | *lws1* | ENSDARG00000044862 | XM_007237519.2 (NCBI) | + | + |
|  | *lws2* | ENSDARG00000044861 | ENSAMXG00000006368 | + | - |
|  | *g101* | N/A | U12024.1 (NCBI) | + | + |
|  |  |  | ENSAMXG00000001701 | - | - |
|  | *g103* | N/A | U12025.1 (NCBI) | + | + |
|  |  |  | ENSAMXG00000001722 | - | - |
| Cone-like non-visual *opsins* | *exo-rod* | ENSDARG00000103574 | ENSAMXG00000017182 | - | + |
|  | *vaa* | KT008402 (NCBI) | ENSAMXG00000009826 | - | - |
|  | *vab* | ENSDARG00000054181 | XM_007259147.2 (NCBI) | - | - |
|  |  |  | ENSAMXG00000010897 | - | - |
|  | *parapinopsina* | KT008404 (NCBI) | XM_007237073.2 (NCBI) | - | - |
|  |  |  | ENSAMXG00000014941 | - | - |
|  | *parapinopsinb* | ENSDARG00000044672 | ENSAMXG00000007169 | - | - |
|  | *parietopsin* | KT008406 | ENSAMXG00000010213 | - | - |
| *tmt / opn3* | *tmtops1a* | ENSDARG00000103674 | ENSAMXG00000016913 | - | - |
|  | *tmtops1b* | ENSDARG00000032246 | ENSAMXG00000008135 | + | + |
|  | *tmtops2a* | KT008407 (NCBI) | KF737856.1 (NCBI) | - | - |
|  | *tmtops2b* | ENSDARG00000027822 | ENSAMXG00000003866 | - | + |
|  |  |  | Transcriptome | - | + |
|  | *tmtops3a* | ENSDARG00000036460 | ENSAMXG00000019922 | + | + |
|  |  |  | Transcriptome | - | + |
|  | *opn3* | ENSDARG00000052775 | XM_007258701.2 (NCBI) | + | + |
|  |  |  | ENSAMXG00000020951 | + | + |
|  |  |  | Transcriptome | + | + |
| *opn6-9* | *opn6a* | ENSDARG00000102430 | ENSAMXG00000020921 | - | - |
|  | *opn6b* | ENSDARG00000098051 | ENSAMXG00000008164 | + | - |
|  |  |  | Transcriptome | + | + |
|  | *opn7a* | ENSDARG00000024208 | ENSAMXG00000002437 | - | - |
|  | *opn7d* | ENSDARG00000068124 | ENSAMXG00000013005 | - | - |
|  | *opn9* | ENSDARG00000104231 | ENSAMXG00000018966 | - | + |
|  |  |  | Transcriptome | - | - |
| *rgr/rrh/opn5* | *opn5* | ENSDARG00000070110 | XM_007239428.1(NCBI) | - | - |
|  |  |  | ENSAMXG00000010179 | - | - |
|  | *rrh* | ENSDARG00000039534 | ENSAMXG00000017584 | - | + |
|  |  |  | Transcriptome | - | + |
|  | *rgra* | ENSDARG00000054890 | ENSAMXG00000012172 | + | + |
|  |  |  | Transcriptome | + | + |
|  | *rgrb* | ENSDARG00000098724 | ENSAMXG00000004323 | + | - |
| *Melanopsins* | *opn4m1* | ENSDARG00000022098 | ENSAMXG00000021230 | + | + |
|  | *opn4m2* | ENSDARG00000007553 | ENSAMXG00000025628 | + | - |
|  | *opn4m3* | ENSDARG00000053929 | ENSAMXG00000001604 | - | - |
|  |  |  | Transcriptome | - | + |
|  | *opn4x1* | ENSDARG00000079129 | ENSAMXG00000006974 | + | - |

Database search by querying gene sequences of the NCBI genbank repository, Ensembl genebuild (Ensembl.org; AstMex 1.0.2, genebuild at July 2016) (McGaugh et al., 2014; Yates et al., 2016), or predicted sequences based on MegaBLAST alignments generated by using NCBI Sequence Read Archive (SRA) (Transcriptome) (see Materials and Methods). +: positive hit in the database search; -: no hit in the database search within the whole adult tissue SRA database (Bioproject ID: PRJNA183542). Each search result per gene was shown if NCBI, Ensembl (ENSAMXG…), or Transcriptome sequences are different, otherwise, search results by using NCBI sequences are shown. *: *A. mexicanus* genome annotation has only one *mws* and no *sws1* at Jan 2018.

**Table S6. Detectable expressions of *A. mexicanus* *opsins* in *A. mexicanus* Databases.**

Database MegaBLAST search against NCBI Sequence Read Archive (SRA) for embryonic to larval stage (from 10 hours-post-fertilization (hpf) to 36 hpf between surface and cavefish; BioProject ID: PRJNA258661) (Stahl and Gross, 2015; Stahl and Gross, 2017; Yoshizawa et al., 2018), and adult surface and cavefish for whole body (BioProject ID: PRJNA183542) (Gross et al., 2013a) (see Materials and Methods). +: positive hit in the database search; -: no hit in the database search by querying annotated sequences (*A. mexicanus* Gene ID) or predicted sequences based on SRA BLAST alignments against zebrafish *opsin* sequences (see Materials and Methods).

**Table S7. Repeated three-way ANOVA results for Figure 6C**

|  | **Statistics** | **P-values** |
| --- | --- | --- |
| Morph (Surface & Cavefish) | F_1,62_ = 34.2 | P < 0.001*** |
| Pinealectomy & Control | F_1,62_ = 0.4 | P = 0.541 |
| Day & Night | F_1,62_ = 36.3 | P < 0.001*** |
| Morph × Pineal&Cont | F_1,62_ = 0.8 | P = 0.382 |
| Morph × Day&Night | F_1,62_ = 13.9 | P < 0.001*** |
| Pineal&Cont × Day&Night | F_1,62_ = 0.5 | P = 0.469 |
| Morph × Pineal&Cont × Day&Night | F_1,62_ = 1.0 | P = 0.325 |

**Table S8. Paired *t*-test results (between Day and Night) after Bonferroni correction for Figure 6C**

|  | **N** | **Statistics** | **P-values** | **P-values after Bonferroni correction** |
| --- | --- | --- | --- | --- |
| Sham-operated control surface fish | 18 | *t*_17_ = 3.0 | P = 0.007 | P = 0.029* |
| Pinealectomized surface fish | 15 | *t*_14_ = 2.1 | P = 0.056 | P = 0.223 |
| Sham-operated control cavefish | 18 | *t*_17_ = 3.9 | P = 0.001 | P = 0.005** |
| Pinealectomized cavefish | 15 | *t*_14_ = 3.8 | P = 0.002 | P = 0.008** |

*: P < 0.05, **: P < 0.01, ***: P < 0.001
